# A comprehensive validation study of the latest version of BoneXpert on a large cohort of Caucasian children and adolescents

**DOI:** 10.3389/fendo.2023.1130580

**Published:** 2023-03-24

**Authors:** Klara Maratova, Dana Zemkova, Petr Sedlak, Marketa Pavlikova, Shenali Anne Amaratunga, Hana Krasnicanova, Ondrej Soucek, Zdenek Sumnik

**Affiliations:** ^1^ Department of Pediatrics, Second Faculty of Medicine, Charles University and Motol University Hospital, Prague, Czechia; ^2^ Department of Anthropology and Human Genetics, Faculty of Science, Charles University, Prague, Czechia; ^3^ Department of Probability and Mathematical Statistics, Faculty of Mathematics and Physic, Charles University, Prague, Czechia

**Keywords:** bone age, Tanner-Whitehouse, Greulich-Pyle, BoneXpert, validation study

## Abstract

**Introduction:**

Automated bone age assessment has recently become increasingly popular. The aim of this study was to assess the agreement between automated and manual evaluation of bone age using the method according to Tanner-Whitehouse (TW3) and Greulich-Pyle (GP).

**Methods:**

We evaluated 1285 bone age scans from 1202 children (657 scans from 612 boys) by using both manual and automated (TW3 as well as GP) bone age assessment. BoneXpert software versions 2.4.5.1. (BX2) and 3.2.1. (BX3) (Visiana, Holte, Denmark) were compared with manual evaluation using root mean squared error (RMSE) analysis.

**Results:**

RMSE for BX2 was 0.57 and 0.55 years in boys and 0.72 and 0.59 years in girls, respectively for TW3 and GP. For BX3, RMSE was 0.51 and 0.68 years in boys and 0.49 and 0.52 years in girls, respectively for TW3 and GP. Sex- and age-specific analysis for BX2 identified the largest differences between manual and automated TW3 evaluation in girls between 6-7, 12-13, 13-14 and 14-15 years, with RMSE 0.88, 0.81, 0.92 and 0.84 years, respectively. The BX3 version showed better agreement with manual TW3 evaluation (RMSE 0.64, 0.45, 0.46 and 0.57).

**Conclusion:**

The latest version of the BoneXpert software provides improved and clinically sufficient agreement with manual bone age evaluation in children of both sexes compared to the previous version and may be used for routine bone age evaluation in non-selected cases in pediatric endocrinology care.

## 1 Introduction

The status of skeletal maturation is the most reliable indicator of biological age in children and adolescents. Bone age (BA) evaluation is a standard procedure widely used in children with growth failure and puberty disturbances. In addition, it is used in chronically ill patients as a complement to overall clinical health assessment (1, 2). BA is used successfully for the timing of orthopedic surgeries in children with uneven length of extremities or specific bone deformities as well.

An x-ray must comprise of the entire hand and wrist to be able to evaluate the bone age. The rationale for this lies in the fact that this skeletal site includes a large number of short bones of which the order and progression of ossification is very well known. Currently, the most common methods of evaluation are the Greulich and Pyle’s method (GP) published in 1959 (3) and the Tanner and Whitehouse 3 method (TW3), where the first edition was published in 1962 (4). While the GP method evaluates the hand as a whole, the TW3 method assigns specific stages of skeletal maturation (1 through 9) to 13 individual pre-determined bones of the hand and wrist (the so-called Radius-Ulna-Short bones, RUS).

Although manual assessment of bone age using both the GP and TW3 methods is reliable if performed by a highly experienced specialist, its main disadvantage is the subjective nature of the procedure. The bone age result of two distinct expert raters may differ by up to a year (5, 6). Thus, automated methods of bone age assessment using software-based morphometric analysis of digitally acquired hand and wrist x-rays have been introduced to clinical practice in the last few years, aiming to eliminate the inherent subjective aspect of the manual work-up and save time. The most sophisticated and currently widely used method of automated bone age analysis works on the platform of the BoneXpert software developed by Visiana (Holte, Denmark). In brief, the software delineates the distal epiphyses of the radius, ulna, metacarpals and phalanges. At least eight bones need to be scored to compute bone age (7). Detailed functioning of the software has been described previously (7, 8).

While the first two commercially released versions of the software already underwent validation with real clinical cases (9, 10), the latest release (issued in 2020) that aimed to improve the limitations of former versions, has not yet been independently tested.

The aims of this study were: 1) to compare manual and automated bone age assessment using BoneXpert software versions BX2 and BX3 using both the GP and TW3 methods in a large cohort of children with various disorders, ages, and sexes, 2) to explore whether the TW3 bone age outcome is affected by differences in the evaluation of individual bones between manual and automated methods.

## 2 Participants and methods

### 2.1 Participants

This cross-sectional retrospective study included 1285 radiographs from 1202 non-selected children and adolescents aged 5 to 16 years (657 scans in 612 boys and 628 scans in 590 girls). All radiographs done for the purpose of bone age assessment at Motol University Hospital between January 2018 and January 2019 were collected. Patients with an abnormal bone structure (e.g. skeletal dysplasia) and patients of non-Caucasian ethnicity^1^ were excluded from the analysis. The software rejected 8 images for poor quality or having an incorrect hand position. Sex-specific one-year age categories were created for girls between 5 and 15 years and boys between 5 and 16 years. Each one-year category included a minimum of 50 radiographs.

This study was approved by the Ethics Committee of the Motol University Hospital (Reference No.: EK-264/18) and complied with the Declaration of Helsinki.

### 2.2 Bone age assessment

After the bone age scan of the left hand and wrist was taken, each image was evaluated manually by one of two experienced raters (M.K. or Z.D.) using both the TW3 (4) and GP (3) methods (only patients sex was disclosed, chronological age was calculated after bone age assessment, diagnosis was not provided to the rater). All images were sent in a standard DICOM format (*Digital Imaging and Communications in Medicine*) for evaluation using automated bone age assessment software BoneXpert (Visiana, Holte, Denmark). No post-processing was applied to the x-rays. The software input consists of patient’s sex, birth date and date of x-ray scan. The BX2 version was used for the purpose of clinical practice, the same images were then reevaluated by the BX3 version as well. This was used only for the purpose of validating the program (the BX3 version was kindly provided by Visiana in form of a StandAlone program for independent evaluation).

If the absolute difference between the manual and automated bone age assessment was more than 1.5 year (an arbitrarily set cut-off in either the GP or TW3 method) the images were reevaluated by an experienced independent rater (S.P.), a medical anthropologist with no affiliation to the Motol University Hospital. An average of the two manual assessments was used for statistical analysis in these cases (N = 70).

### 2.3 Statistical analysis

Throughout the analysis, repeated measurements on the same child were treated as independent observations as they were gained at different visits.

The Bland-Altman analysis was used to determine the character of differences between the automated and manual approach. For each patient, Bland-Altman plots the difference between the automated and manual assessment against the mean of the two methods, or alternatively, against the values of one of the two methods. In this analysis, differences were plotted against the results of the manual method. The graphs indicate where the automated method produced higher or lower values in comparison to the manual method, possible bias (mean of differences) and lower and upper limit of accuracy (LOA), computed as bias ± 2×standard deviation (SD). Bias of each method was tested using a one-sample t-test, the bias between BX2 and BX3 were compared using paired t-tests.

To explore the size of differences between manual and automated bone age assessment in general and in various categories (defined by sex and/or age and diagnosis), Root Mean Squared Errors (RMSE) were calculated using the standard formula (12):

$$\text{RMSE}={\left[\sum_{\text{i}=1}^{\text{N}}{\left({\text{z}}_{fi}-{\text{z}}_{oi}\right)}^{2}/\text{N}\right]}^{1/2}$$

where:

- Σ = summation
- (z*_fi_*-z*_oi_*)^2^ = differences, squared
- N = sample size

Confidence intervals for RMSE were computed under the assumption of symmetry of deviations of BoneXpert estimates compared to manual assessment. Accuracy of BX2 with respect to manual assessment was compared to the accuracy of BX3 with respect to manual assessment using the Diebold-Mariano test (13).

In the detailed analysis of the TW3 method, the difference between stages assigned by manual and automated method were compared using ANOVA F-test and *post-hoc* pairwise comparisons with Benjamini-Hochberg correction for multiple comparisons. The differences in assigned bone stages were tested in all available scans divided into 3 groups according to the difference in the final bone age (BX higher than manual by >1.0 year; BX lower than manual by > 1.0 year; BX not different from manual, i.e.<1.0 and > - 1.0 year). In bones showing the greatest differences in assigned bone stages, the effect on resulting bone age was tested.

All analyses were performed in statistical language and environment R, version 4.1.2 (14). The level of statistical significance was set to 0.05 throughout the analysis. In case of multiple comparisons adjustment (such as testing in various age-, sex- or diagnosis-specific categories), the Benjamini-Hochberg method was used.

## 3 Results

### 3.1 Comparison between automated and manual bone age assessment in children according to sex and age

Using the TW3 method, the BX2 version generally underestimated bone age in both sexes, whereas the BX3 version performed comparably to the manual assessment with mean of the differences close to zero (**Table 1** – the data are given in years). On the other hand, BX3 performed significantly worse using the GP method compared to BX2 version in boys (**Table 1**). In particular, while BX2-assessed GP bone age did not differ from manually assessed GP bone age in boys, the BX3 version significantly overestimated GP bone ages. In girls, both BX2 and BX3 slightly underestimated GP bone age compared to manual evaluation.

**Table 1:** Overall means of differences in years between automated and manual bone age assessment, separately for both sexes and software versions (BX2 and BX3).

		TW3				GP			
	N	BX2 – MAN		BX3 – MAN		BX2 – MAN		BX3 – MAN	
		mean (SD)	P	mean (SD)	P	mean (SD)	p	mean (SD)	P
Boys	657	-0.19 (0.54)	< 0.0001	-0.01 (0.51)	0.239	-0.00 (0.55)	0.924	0.39 (0.56)	< 0.0001
Girls	628	-0.47 (0.55)	< 0.0001	-0.02 (0.49)	0.635	-0.23 (0.55)	< 0.0001	-0.10 (0.51)	< 0.0001

P-values for one-sample t-test examining the difference from zero.

TW3, bone age assessment according to Tanner-Whitehouse 3 method; GP, bone age assessment according to Greulich-Pyle method; BX2, BoneXpert version 2.4.5.1.; BX3, BoneXpert version 3.0.3.; MAN, manual bone age assessment.

**Figure 1:**
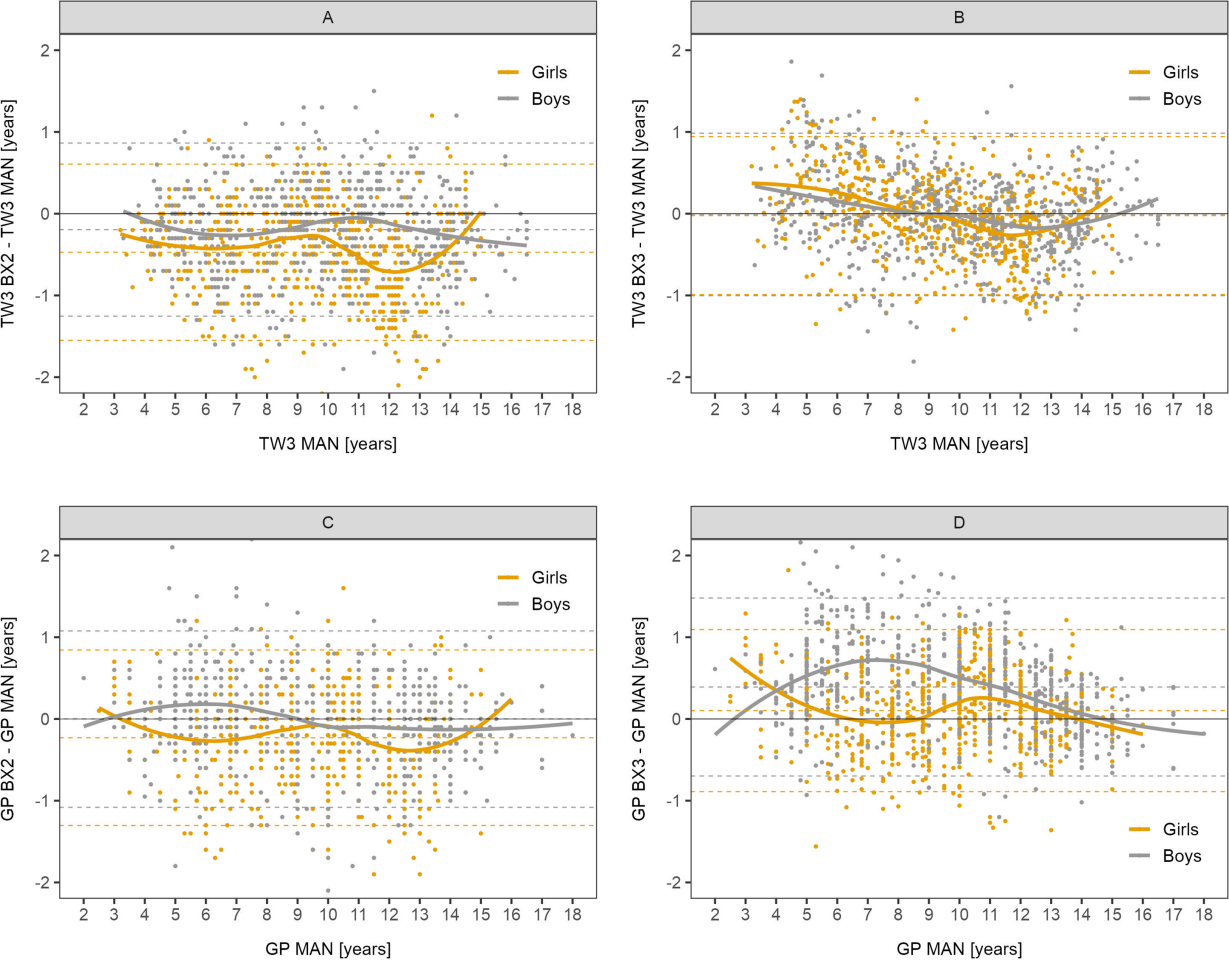
Bland-Altman analysis – difference in years between automated and manual bone age result plotted against the manual bone age values. Sex-specific smoothing lines computed by LOESS method. Bland-Altman analysis shows whether there is a systematic component to the differences between methods. Dashed lines show mean and upper and lower limit of accuracy for respective methods and sex. The closer the mean to 0 the less over/underestimating the method is, overall. **(A)** TW3, BX2 vs. manual, **(B)** TW3, BX3 vs. manual, **(C)** GP, BX2 vs. manual, **(D)** GP, BX3 vs. manual. TW3, bone age assessment according to Tanner-Whitehouse 3 method; GP, bone age assessment according to Greulich-Pyle method; BX2, BoneXpert version 2.4.5.1.; BX3, BoneXpert version 3.0.3.; MAN, manual bone age assessment.

The differences between automated and manual bone age results are presented in detail in Bland-Altman graphs in **Figure 1**. The best agreement was observed in the BX3 version using the TW3 method in both sexes (**Figure 1B**).

These findings were further supported by the RMSE analysis showing that the BX3 version has significantly better agreement with manual bone age assessment than the BX2 version in both sexes using the TW3 method and in girls using the GP method as well (**Table 2** - the data are given in years). In contrast, the BX3 version performed worse than BX2 in boys using the GP method.

**Table 2:** Root mean square errors of automated vs. manual bone age assessment, separately for both sexes and software versions (BX2 and BX3).

		TW3			GP		
	N	BX2 vs MAN	BX3 vs MAN	p	BX2 vs MAN	BX3 vs MAN	P
Boys	657	0.57 (0.54-0.61)	0.51 (0.48-0.54)*	0.0007	0.55 (0.52-0.58)	0.68 (0.64-0.72) #	< 0.0001
Girls	628	0.72 (0.69-0.77)	0.49 (0.47-0.52)*	< 0.0001	0.59 (0.56-0.63)	0.52 (0.49-0.55)*	< 0.0001

Root mean square errors (and corresponding 95% confidence intervals) are shown (in years).

p-value: Diebold-Mariano test for method accuracy (* BX3 performs significantly better than BX2 α= 0.05, # BX3 performs significantly worse than BX2 at α =0.05).

TW3, bone age assessment according to Tanner-Whitehouse 3 method, GP, bone age assessment according to Greulich-Pyle method, BX2, BoneXpert version 2.4.5.1., BX3, BoneXpert version 3.0.3., MAN, manual bone age assessment.

**Figure 2:**
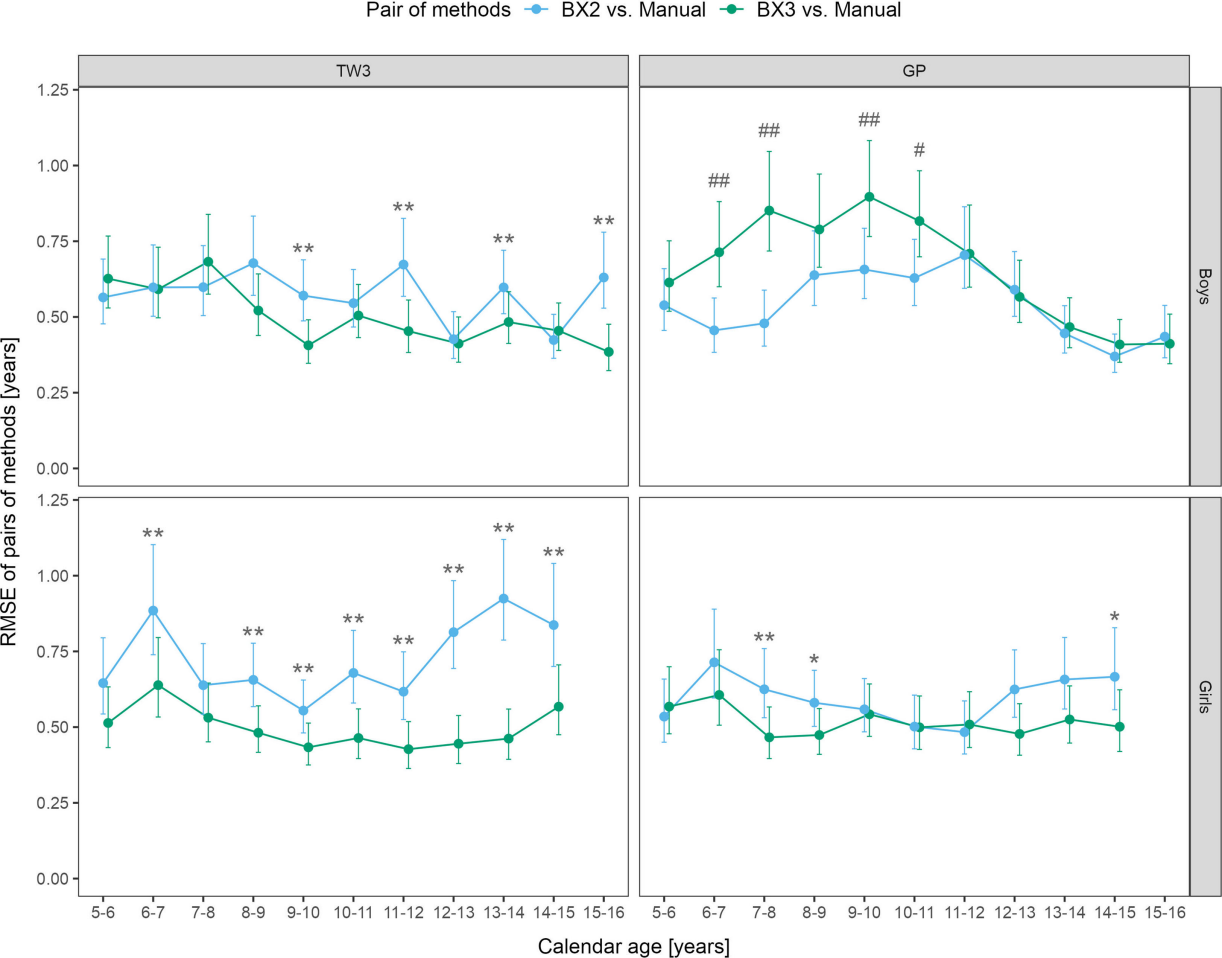
Root mean square errors (RMSE) and 95% confidence intervals for age and sex specific categories, separately for TW3 and GP methods and for BX2 and BX3 versions. RMSE is given in years. Before (*) and after (**) adjustment for multiple testing, BX3 performs significantly better, i.e. differs less from the manual assessment, than BX2 at α = 0.05. Before (#) and after (##) adjustment for multiple testing, BX3 performs significantly worse, i.e. differs more from the manual assessment, than BX2 at α =0.05. TW3, bone age assessment according to Tanner-Whitehouse 3 method; GP, bone age assessment according to Greulich-Pyle method; BX2, BoneXpert version 2.4.5.1.; BX3, BoneXpert version 3.0.3.; MAN, manual bone age assessment.

Sex- and age-specific RMSE for the BX2 version using the TW3 method showed that the largest differences between automated and manual bone age were present in girls aged 6-7 and 12-15 years (**Figure 2**). When using the BX3 version, the agreement between automated and manual bone age improved significantly in 8/10 age categories in girls, when compared to BX2. For the GP method, BX2 showed significantly larger RMSE than the BX3 version only in girls aged 7-8 years.

In boys, the BX3 version showed improvement of the TW3 method in 4 age categories (9-10, 11-12, 13-14 and 15-16 years), compared to BX2 (**Figure 2**). In contrast, the RMSEs between manual and automated bone age evaluation were larger when using the BX3 version compared to BX2 using the GP method in boys, in particular for ages 6-8 and 9-10 years. The RMSE numeric values (in years) are presented in **Supplementary Table 1**.

The absolute difference in bone age result > 1.0 year was noted in 7.5% and 6.2% scans in boys and 16.4% and 8.4% scans in girls, for TW3 and GP respectively, when using the BX2 version. The BX3 version showed > 1.0 year difference in 6.3% and 12.8% scans in boys and 6.0% and 5.3% scans in girls for TW3 and GP, respectively.

### 3.2 Agreement between automated and manual bone age assessment in children with various diagnoses

**Figure 3:**
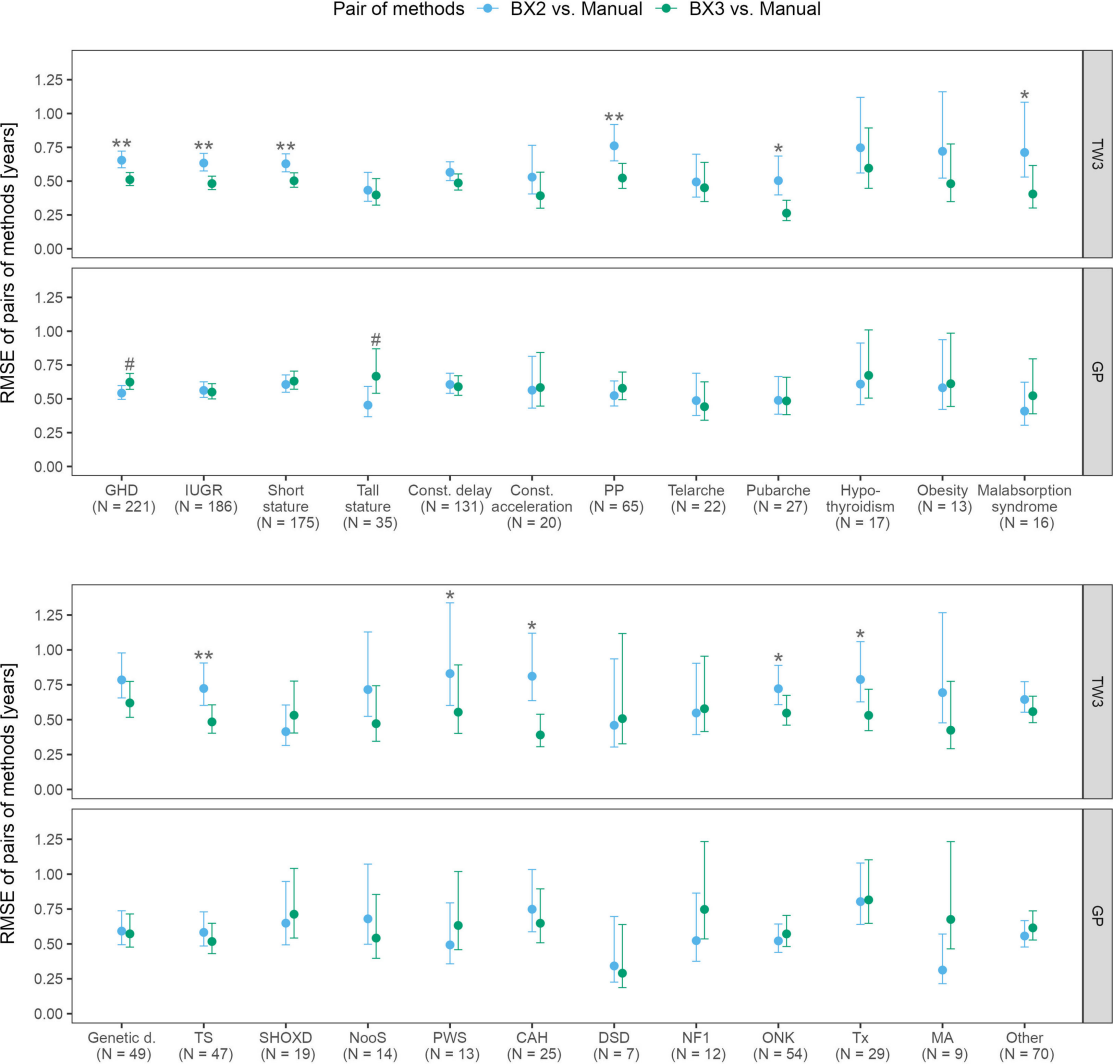
Root mean square errors (RMSE) and 95% confidence intervals for various diagnoses, separately for TW3 and GP methods and for both software versions (BX2 and BX3). RMSE is given in years. Before (*) and after (**) adjustment for multiple testing, BX3 performs significantly better, i.e. differs less from the manual assessment, than BX2 at α = 0.05, Diebold-Mariano test for method accuracy. Before (#) and after (##) adjustment for multiple testing, BX3 performs significantly worse, i.e. differs more from the manual assessment, than BX2 at a =0.05, Diebold-Mariano test for method accuracy. TW3, bone age assessment according to Tanner-Whitehouse 3 method; GP, bone age assessment according to Greulich-Pyle method; BX2, BoneXpert version 2.4.5.1.; BX3, BoneXpert version 3.0.3.; MAN, manual bone age assessment. GHD, growth hormone deficiency; IUGR, intra-uterine growth restriction; Const. delay, constitutional delay of growth; Const. acceleration, constitutional acceleration of growth; PP, precocious puberty; Genetic d., genetic disorders; TS, Turner syndrome; SHOXD, SHOX gene deficiency (all patients were treated with growth hormone); NooS, Noonan syndrome; CAH, congenital adrenal hyperplasia (16/25 were diagnosed with classical CAH); DSD, disorders of sex differenciation; NF1, neurofibromatosis type 1; ONK, oncology disorders; Tx, patients after liver; kidney or bone marrow transplant, MA, anorexia nervosa.

The RMSE analysis confirmed that the best agreement between automated and manual bone age evaluation was reached when using the TW3 method in BX3, regardless of the patient’s disease (**Figure 3**). Disease-specific RMSEs are shown in **Supplementary Table 2**.

The disease specific mean differences between automated and manual bone age values showed that the TW3 BX2 bone age differed significantly from manual evaluation in 16/24 disease groups. BX3 showed significant improvement, only children with growth hormone deficiency differed significantly from manual testing. The particular differences given in years are shown in **Supplementary Figure 1**.

### 3.3 Detailed analysis of the TW3 method: Differences of the automated and manual evaluation of particular bones and the effect on the outcome of the final bone age

A detailed analysis of the TW3 method was carried out on 1206 scans with detailed data on individual bones available. Out off these, 145 BX2 assessments (12.0%) differed by more than 1 year from the manual assessment, most of these (139) being lower than the manually estimated bone age. Seventy-four BX3 assessments (6.1%) differed by more than 1 year from the manual assessment (while being much more equally distributed: 47 were lower and 27 higher than the manually assessed bone age).

For each automated bone age software version and each group according to whether automated assessment resulted in the bone age being 1) > 1.0 year higher, 2) >1.0 year lower, or 3) less than one year different from the manually assessed bone age, differences in individual bone scores for each of the 13 bones were examined graphically (**Supplementary Figure 2**) and by using the ANOVA method with *post-hoc* pairwise comparisons. Out of these radius and ulna showed larger differences in assigned bone score among other bones (ANOVA F-test p< 0.001).

**Figure 4:**
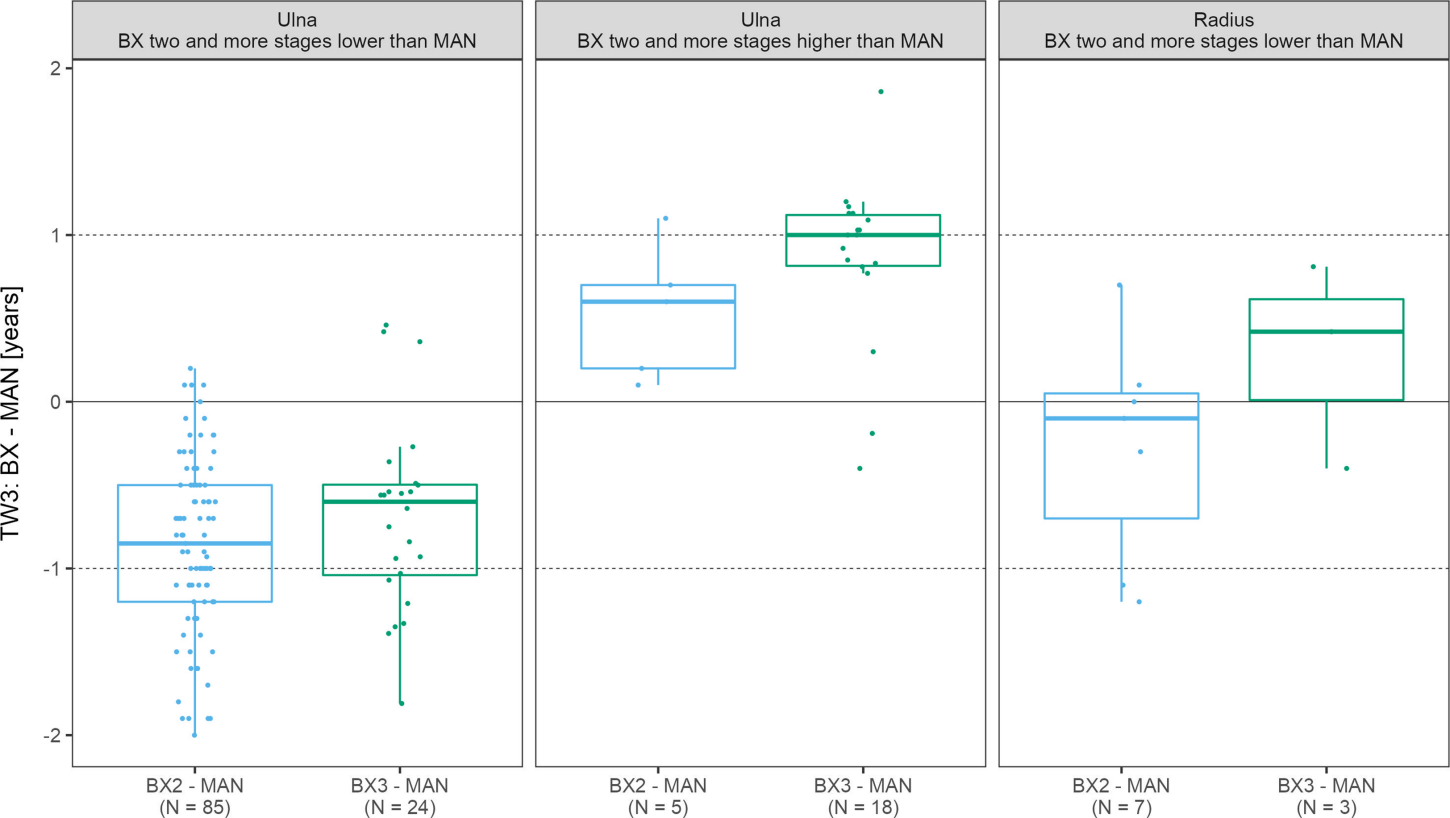
The boxplots depict the distribution and mean differences in years between automated and manual final bone age in scans where bone stage assigned to radius/ulna exceeded 2 stages. TW3, bone age assessment according to Tanner-Whitehouse 3 method; GP, bone age assessment according to Greulich-Pyle method; BX2, BoneXpert version 2.4.5.1.; BX3, BoneXpert version 3.0.3.; MAN, manual bone age assessment.

While focusing only on those x-rays where the ulna and/or radius scoring differed by more than 1 stage between automated and manual assessment,we have identified 90 such scans for the ulna with the BX2 version (85 underestimated and 5 overestimated scores) and 42 scans with BX3 (24 underestimated and 18 overestimated scores). For the radius, there were only 7 and 0 cases for BX2 and 3 and 0 cases for BX3, with under- and overestimated scores, respectively. In scans where BX3 under- or over-estimated the evaluation of the ulna, the mean difference between the automated (BX3) and manual bone age deviated significantly from 0 (p< 0.001) however the mean difference did not exceed 1 year (**Figure 4** and **Supplementary Table 3**). The absolute difference in bone age exceeded 1 year (N = 15; median absolute difference 1.2 years; IQR 1.1-1.3 years) only in a minority of these cases and there was no discernable pattern in sex or diagnoses.

## 4 Discussion

The objective of this study was to explore the clinical utility of the BoneXpert automated bone age assessment on a large unselected cohort of children. We showed that the latest BoneXpert version (BX3) performed comparably to expert manual bone age reading in a large cohort of Caucasian children and that it performed better than the previous BoneXpert version (BX2). In particular, BX2-inherent underestimation of TW3 bone age, which was more pronounced in girls, was completely abolished in the newer BX3 version. The TW3 bone age assessed by the BX3 performed best among myriad of diseases as well, in which bone age is typically evaluated. Thus, this study encourages the use of automated TW3 bone age assessment in daily clinical practice.

Validation of automated bone age assessment is typically done by comparing the result to bone age assessed manually by a highly experienced individual. We showed that the BX2 version underestimated TW3 bone age especially in girls aged 6 to 7 and 12 to 15 years, when compared to manually-assessed TW3 bone age. Our results were similar to a previous study in participants of the First Zurich Longitudinal Study, where the differences between automated and manual TW3 bone age assessment (RMSEs) were reported to be 0.67 years in boys and 0.63 years in girls (10). The authors (10) noted considerable variability between individual age categories but did not show the data in extenso. Interestingly, our study showed that this inherent limitation of the BX2 version has been abolished in the latest software version (BX3).

There are no studies published comparing the TW3 bone age outcome between BX2 and BX3, only a single previous study explored the performance of the first (BX1) and third (BX3) software versions with regard to GP bone age (8, 15). In the Caucasian population a RMSE of 0.66 and 0.51 years in boys and 0.50 and 0.48 years in girls was reported, for BX1 and BX3 respectively. This was similar to our study, in which the BX3 version of GP bone age differed from the manual rating by 0.68 and 0.52 years in boys and girls respectively. Interestingly the GP results reported by Martin et al. (8) were in significantly worse agreement in girls of African descent (RMSE 0.75 years). On the other hand, a similar study on children of Indian ethnicity found the agreement between manual and automated GP bone age in girls to be 0.39 years (RMSE) (16). As both GP and TW3 methods are based on the Caucasian population, the causes are probably the differences in skeletal maturation among different ethnicities, geographical location and socioeconomic status (8, 17, 18) - in the Czech Republic the agreement between sexual maturation and bone age provided by the GP and TW3 methods has been well established (19).

To enhance clinical utility, automated bone age analysis needs proper validation in individual diseases. The BoneXpert software was introduced in 2009 (7) and the agreement of the first version with GP manual rating has been evaluated in children with a few common endocrine disorders (20–22). Our study explored the agreement between automated and manual bone age assessment in a large unselected group of disorders that can be commonly encountered in pediatric clinical practice. We showed that the BX3 version TW3 method performs consistently across various disorders. Interestingly, the RMSE for the TW3 method of the BX3 version were lower than the RMSE for GP in the first version of the software (22) in children with growth hormone deficiency or Turner syndrome (0.50 vs. 0.71 and 0.48 vs. 0.75, respectively). These results further support the use of the latest TW3 BoneXpert version in clinical practice.

In every automated analysis algorithm, systemic scoring errors should be excluded to avoid improper bone age assessment. The automated TW3 assessment by BoneXpert displays the scoring of individual bones, which allows for a more in-depth analysis. We showed that automated ulna scoring resulted in larger differences from the manual scores compared to the other bones. However, this did not have a significant influence on the TW3 bone age value. This eliminates the possibility that the differences between automated and manual TW3 bone age values may be due to systemic errors in the evaluation of a particular bone.

The strengths of this study are: 1) the large cohort of patients of Caucasian descent with various disorders, representing the common clinical situation, in whom we validated the latest version of automated GP as well as TW3 bone age assessment provided by BoneXpert, 2) the direct comparison between the latest software version (BX3) and the previous widely used version (BX2) and 3) the in depth analysis of the TW3 method.

As a limitation of this study we recognize: 1) the homogeneous cohort of children with Caucasian descent, therefore we recommend caution when applying our results to the non-Caucasian population, 2) that the disease-specific RMSEs were not further analyzed with regard to sex. This was due to relatively low number of children in certain groups with rare disorders and because we found no statistically significant difference between boys and girls in the overall RMSE analysis of the TW3 BX3 version.

The strengths of BoneXpert software include: 1) time efficiency - the number of specialists that spent more than 2 minutes evaluating an image decreased from 86 to 21% after installation of BoneXpert (23), 2) ease of use, 3) validation in different ethnicities (15) and various disorders (20–22), and 4) wide use (8). On the other hand 1) cost effectiveness in lower income countries may be an issue and 2) precision was not yet established.

## 5 Conclusion

Bone age analysis provided by the most recent BoneXpert software version showed clinically reliable agreement with manual evaluation among wide range of chronic diseases of children. BoneXpert is therefore a good alternative to manual rating. There are few relevant clinical implications for the use of BoneXpert in clinical practice. The major advantage is the ability to save time of the experienced evaluators. Manual bone age analysis could thus be reserved for cases where automated analysis performs improbably (i.e., discrepancy between bone age and sexual maturation) or is not feasible (i.e., skeletal dysplasia).On the other hand, bone morphology and structure, besides the bone age assessment, is routinely evaluated as part of the manual workup. The automated system does not provide such a feature. Thus, patients with mild to moderate skeletal dysplasia (which is clinically discrete) may escape the appropriate medical attention.

## Data availability statement

All data generated and analyzed in this study are available from the corresponding author on reasonable request.

## Ethics statement

The studies involving human participants were reviewed and approved by the Ethics Committee of the Motol University Hospital (Reference No.: EK-264/18). Written informed consent from the participants’ legal guardian/next of kin was not required to participate in this study in accordance with the national legislation and the institutional requirements.

## Author contributions

KM and DZ contributed equally to the conception, design and data collection. PS performed the independent reevaluation of selected bone age scans. MP performed the statistical analysis. KM wrote the draft of the manuscript and ZS, OS, HK and SA were involved in data analysis and editing of the manuscript. All authors contributed to the article and approved the submitted version.

## Footnotes

1 According to the 2021 census, the Czech population is homogeneous, the largest minority is of Vietnamese descent and makes up only 0.4% of the population (11).
