# Supplementary material for: A comprehensive validation study of the latest version of BoneXpert on a large cohort of Caucasian children and adolescents
Source: Front Endocrinol (Lausanne). 2023 Mar 24;14:1130580. doi: 10.3389/fendo.2023.1130580 (PMC10079872; doi:10.3389/fendo.2023.1130580)
Supplement: Supplementary file 1 [file DataSheet_1.pdf]

Supplementary Material

A Comprehensive Validation Study of BoneXpert latest version on a Large Cohort of Caucasian Children and Adolescents

Maratova Klara*, Zemkova Dana, Sedlak Petr, Pavlikova Marketa, Amaratunga Shenali Anne, Krasnicanova Hana, Soucek Ondrej, Sumnik Zdenek

***Correspondence:**
Klara Maratova, Ph.D.
klara.maratova@fnmotol.cz

1 Supplementary Tables

Table 1: Differences between manual and two versions of software for automated bone age assessment (BX2 and BX3) in sex-dependent one-year age categories.

	Boys								Girls							
**Age**	**TW3**				**GP**				**TW3**				**GP**			
**categories**	**MAN vs BX2**	**MAN vs BX3**	**p**	**p adjust.**	**MAN vs BX2**	**MAN vs BX3**	**p**	**p adjust.**	**MAN vs BX2**	**MAN vs BX3**	**p**	**p adjust.**	**MAN vs BX2**	**MAN vs BX3**	**p**	**p adjust.**
5-6	0.56	0.63	0.441	0.617	0.54	0.61	0.320	0.481	0.65	0.51	0.217	0.351	0.53	0.57	0.628	0.768
6-7	0.60	0.59	0.929	0.972	0.46	0.71	**<0.001**	**0.005**	0.88	0.64	**0.007**	**0.019**	0.71	0.61	0.159	0.267
7-8	0.60	0.68	0.261	0.406	0.48	0.85	**<0.001**	**<0.001**	0.64	0.53	0.143	0.250	0.62	0.47	**0.006**	**0.018**
8-9	0.68	0.52	0.079	0.144	0.64	0.79	**0.055**	0.110	0.66	0.48*	**0.004**	**0.014**	0.58	0.47	**0.022**	**0.054**
9-10	0.57	0.41	**0.002**	**0.009**	0.66	0.90	**0.002**	**0.008**	0.55	0.43	**0.008**	**0.022**	0.56	0.54	0.640	0.768
10-11	0.55	0.50	0.494	0.670	0.63	0.82	**0.035**	0.078	0.68	0.46	**0.001**	**0.005**	0.50	0.50	0.966	0.972
11-12	0.67	0.45	**0.004**	**0.014**	0.70	0.71	0.972	0.972	0.61	0.43	**0.001**	**0.005**	0.48	0.51	0.610	0.768
12-13	0.43	0.41	0.742	0.800	0.59	0.57	0.701	0.796	0.81	0.45	**<0.001**	**<0.001**	0.62	0.48	0.058	0.110
13-14	0.60	0.48	**0.003**	**0.013**	0.45	0.47	0.739	0.800	0.92	0.46	**<0.001**	**<0.001**	0.66	0.53	0.052	0.109
14-15	0.42	0.45	0.559	0.733	0.37	0.41	0.382	0.553	0.84	0.57	**<0.001**	**0.003**	0.67	0.50	**0.033**	0.076
15-16	0.63	0.38	**<0.001**	**0.003**	0.44	0.41	0.666	0.777	-	-	-	-	-	-	-	-

Root mean square errors are shown (in years).

p-values: p = Diebold-Mariano test for method accuracy, p adjust = p values adjusted for multiple comparisons using Benjamini-Hochberg method

TW3 = bone age assessment according to Tanner-Whitehouse 3 method, GP = bone age assessment according to Greulich-Pyle method, BX2 = BoneXpert version 2.4.5.1., BX3 = BoneXpert version 3.0.3., MAN = manual bone age assessment

Table 2: The differences between manual and automated bone age assessment in patients according to the diagnosis.

Diagnosis (N)	TW3				GP			
	MAN vs BX2	MAN vs BX3	P	p adjusted	MAN vs BX2	MAN vs BX3	p	p adjusted
Growth hormone deficiency (221)	0.65	0.51	**<0.001**	**0.003**	0.54	0.62	**0.014**	0.068
Intra-uterine growth restriction (186)	0.63	0.48	**<0.001**	**0.003**	0.56	0.55	0.764	0.837
Short stature (175)	0.63	0.50	**0.003**	**0.041**	0.61	0.63	0.608	0.775
Tall stature (35)	0.43	0.40	0.565	0.775	0.45	0.67	**0.022**	0.089
Constitutional delay (131)	0.57	0.49	0.103	0.291	0.61	0.59	0.746	0.837
Constitutional acceleration (20)	0.53	0.39	0.077	0.247	0.56	0.58	0.845	0.902
Precocious puberty (65)	0.76	0.52	**<0.001**	**0.015**	0.52	0.58	0.454	0.708
Telarche (22)	0.49	0.45	0.613	0.775	0.49	0.44	0.610	0.775
Pubarche (27)	0.50	0.26	**0.012**	0.068	0.49	0.48	0.958	0.978
Hypothyroidism (17)	0.75	0.60	0.338	0.639	0.61	0.67	0.687	0.837
Obesity (13)	0.72	0.48	0.307	0.614	0.58	0.61	0.767	0.837
Malabsorption syndrome (16)	0.71	0.40	**0.027**	0.101	0.41	0.52	0.493	0.740
Genetic disorder (49)	0.79	0.62	**0.057**	0.195	0.59	0.57	0.745	0.837
Turner syndrome (47)	0.72	0.48	**0.005**	**0.047**	0.58	0.52	0.277	0.604
SHOX deficiency (19)	0.41	0.53	0.294	0.613	0.65	0.71	0.438	0.708
Noonan syndrome (14)	0.72	0.47	0.086	0.259	0.68	0.54	0.239	0.574
Prader-Willi syndrome (13)	0.83	0.55	**0.018**	0.077	0.49	0.63	0.567	0.775
Congenital adrenal hypertrophy (25)	0.81	0.39	**0.009**	0.068	0.75	0.65	0.373	0.663
Disorders of sex development (7)	0.46	0.51	0.957	0.978	0.34	0.29	0.606	0.775
Neurofibromatosis type 1 (12)	0.55	0.58	0.721	0.837	0.52	0.75	0.151	0.382
Oncology disorders (54)	0.72	0.55	**0.013**	0.068	0.52	0.57	0.457	0.708
Kidney, liver or bone marrow transplant (29)	0.79	0.53	**0.011**	0.068	0.80	0.82	1.000	1.000
Anorexia nervosa (9)	0.69	0.42	0.346	0.639	0.31	0.68	0.109	0.291
Other (70)	0.64	0.56	0.269	0.604	0.56	0.62	0.407	0.698

Root mean square errors are shown (in years).

p-values: p = Diebold-Mariano test for method accuracy, p adjust = p values adjusted for multiple comparisons using Benjamini-Hochberg method

* TW3 = bone age assessment according to Tanner-Whitehouse 3 method, GP = bone age assessment according to Greulich-Pyle method, BX2 = BoneXpert version 2.4.5.1., BX3 = BoneXpert version 3.0.3., MAN = manual bone age assessment

Table 3: The differences between automated and manual bone age in scans where bone stage assigned to radius/ulna exceeded 2 stages.

Situation	N	mean	SD	95 % CI	p
Ulna BX2 two and more stages lower than MAN	85	-0.863	0.529	(-0.98, -0.75)	< 0.001
Ulna BX2 two and more stages higher than MAN	5	0.540	0.404	(0.04, 1.04)	0.040
Radius BX2 two and more stages lower than MAN	7	-0.271	0.675	(-0.89, 0.35)	0.328
Ulna BX3 two and more stages lower than MAN	24	-0.684	0.567	(-0.92, -0.45)	< 0.001
Ulna BX3 two and more stages higher than MAN	18	0.863	0.516	(0.61, 1.12)	< 0.001
Radius BX3 two and more stages lower than MAN	3	0.277	0.618	(-1.26, 1.81)	0.519

Means with standard deviation (SD) of differences between automated and manual assessment. Given p-value corresponds to t-test for zero bias.

BX2 = BoneXpert version 2.4.5.1., BX3 = BoneXpert version 3.0.3., MAN = manual bone age assessment

2 Supplementary Figures

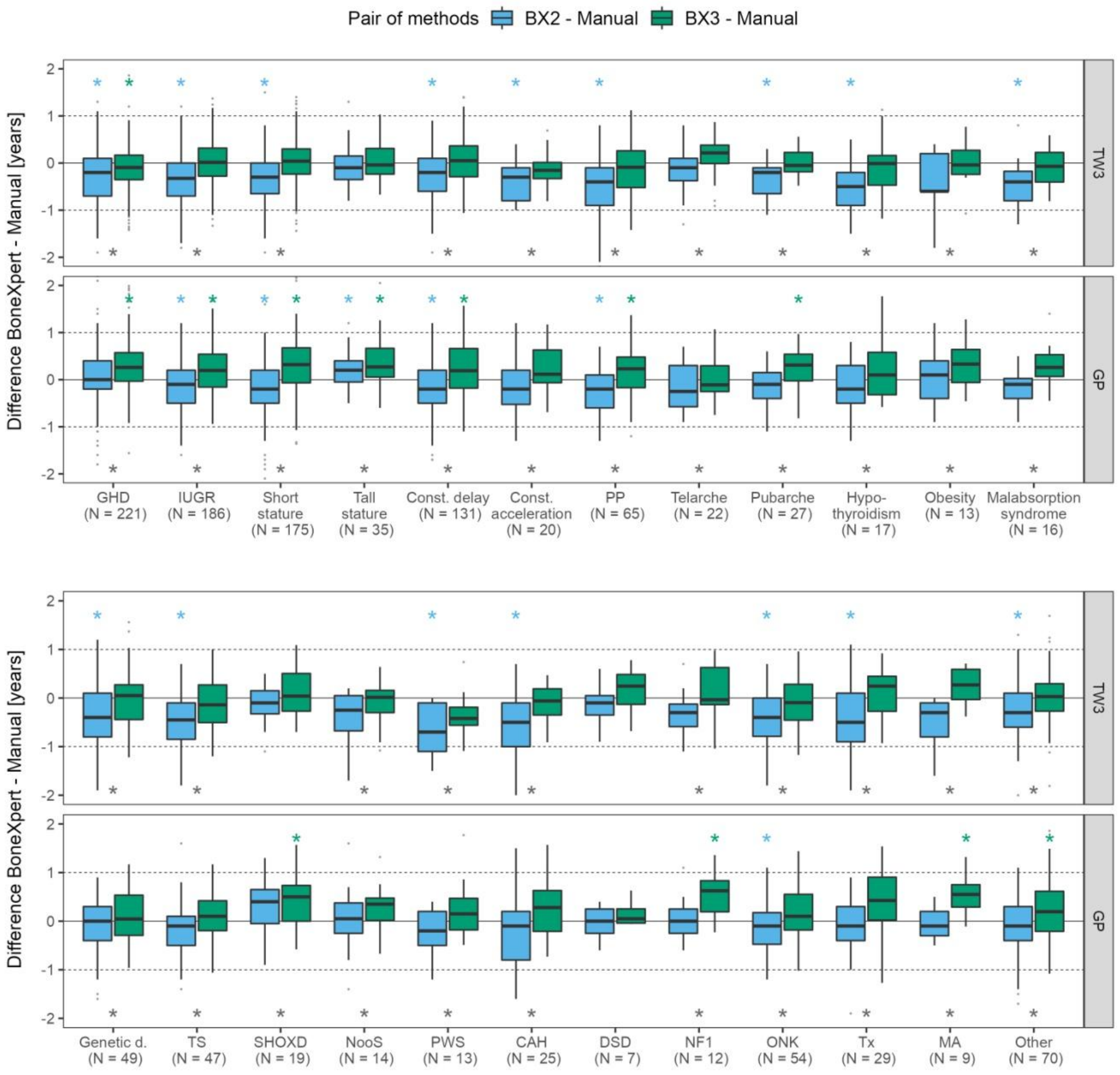

Figure 1: Boxplots of differences in years between automated and manual bone age assessment for various diagnoses, separately for TW3 and GP methods and for both software versions (BX2 and BX3)

Blue (*): BX2 differs significantly from manual at α = 0.05 (no zero bias, one-sided t-test, adjusted for multiple testing).

Green (*): BX3 differs significantly from manual at α = 0.05 (no zero bias, one-sided t-test, adjusted for multiple testing).

Gray (*) Bias of BX2 and bias of BX3 are significantly different at α = 0.05 (paired t-test, adjusted for multiple testing).

TW3 = bone age assessment according to Tanner-Whitehouse 3 method, GP = bone age assessment according to Greulich-Pyle method, BX2 = BoneXpert version 2.4.5.1., BX3 = BoneXpert version 3.0.3., MAN = manual bone age assessment

GHD = growth hormone deficiency, IUGR = intra-uterine growth restriction, Const. delay = constitutional delay of growth, Const. acceleration = constitutional acceleration of growth, PP = precocious puberty, Genetic d. = genetic disorders, TS = Turner syndrome, SHOXD = SHOX gene deficiency (all patients were treated with growth hormone), NooS = Noonan syndrome, CAH = congenital adrenal hyperplasia (16/25 were diagnosed with classical CAH), DSD = disorders of sex differenciation , NF1 = neurofibromatosis type 1 , ONK = oncology disorders , Tx = patients after liver, kidney or bone marrow transplant, MA = anorexia nervosa

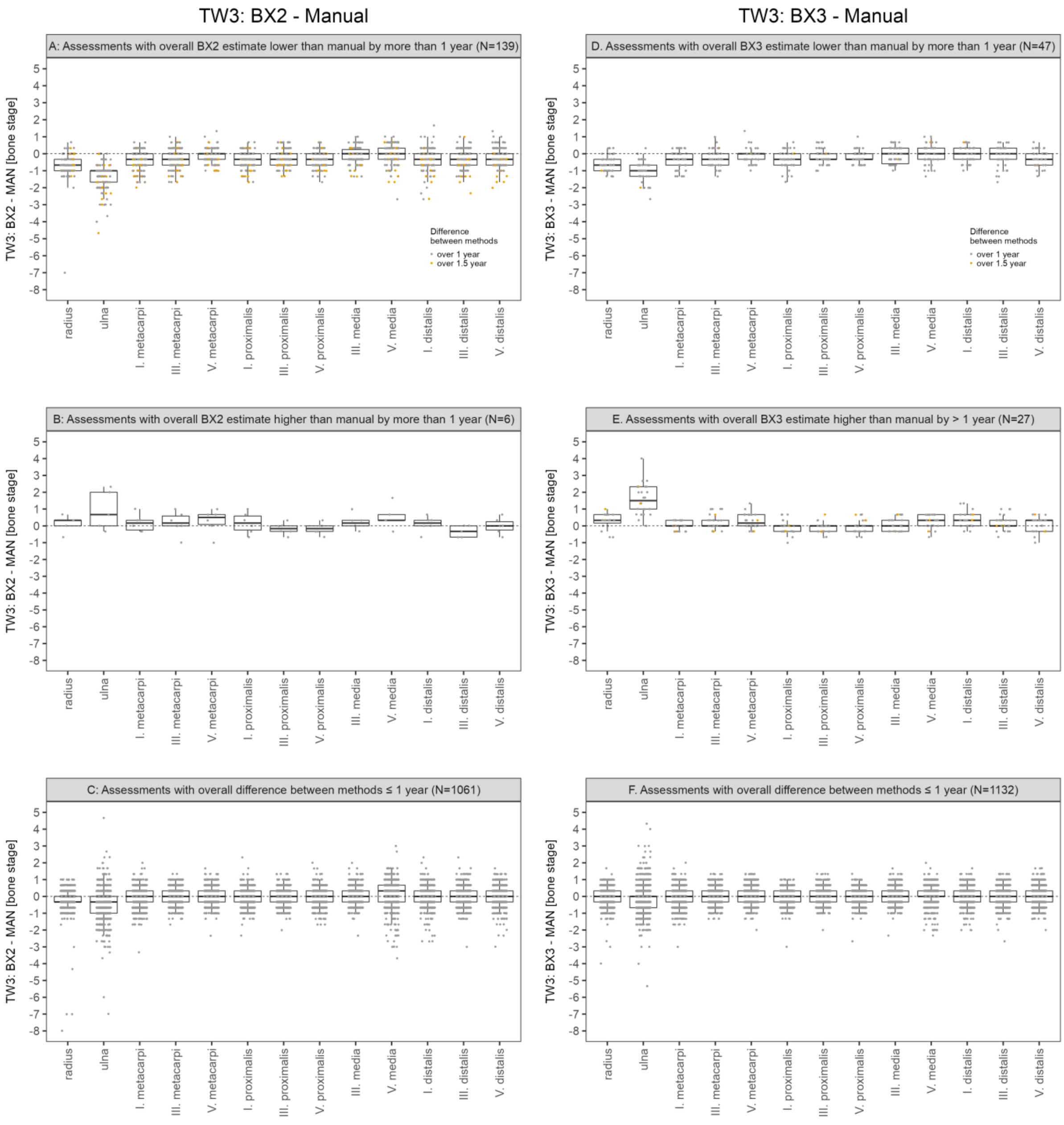

Figure 2: The variability in stages assigned to individual bones when using the TW3 method; BX2 vs. manual (A-C) and BX3 vs. manual (D-F).

The difference is defined as “BoneXpert - manual” (positive difference means that the BoneXpert determined stage is higher). Individual values and summarizing boxplots are plotted. Assessments are segregated by the overall difference in assessed bone age: in A+ D manual dominates BX by more than 1 year, in B+E BX dominates manual by more than 1 year, in C+F the methods differ by less than 1 year.
